# Important functional role of the protein osteopontin in the progression of malignant pleural mesothelioma

**DOI:** 10.3389/fimmu.2023.1116430

**Published:** 2023-06-16

**Authors:** Elisabeth Digifico, Marco Erreni, Laura Mannarino, Sergio Marchini, Aldo Ummarino, Clément Anfray, Luca Bertola, Camilla Recordati, Daniela Pistillo, Massimo Roncalli, Paola Bossi, Paolo Andrea Zucali, Maurizio D’Incalci, Cristina Belgiovine, Paola Allavena

**Affiliations:** ^1^ Department Immunology, IRCCS Humanitas Research Hospital, Milan, Italy; ^2^ Unit of Advanced Optical Microscopy, IRCCS Humanitas Research Hospital, Milano, Italy; ^3^ Lab. Cancer Pharmacology, IRCCS Humanitas Research Hospital, Milano, Italy; ^4^ Department Biomedical Sciences, Humanitas University, Milano, Italy; ^5^ Mouse and Animal Pathology Lab., Fondazione Unimi, and Department of Veterinary Medicine and Animal Sciences, University of Milano, Lodi, Italy; ^6^ Biobank, Humanitas IRCCS Humanitas Research Hospital, Milano, Italy; ^7^ Department Pathology, IRCCS Humanitas Research Hospital, Milan, Italy; ^8^ Department Oncology, IRCCS Humanitas Research Hospital, Milano, Italy

**Keywords:** osteopontin (OPN), MPM (malignant pleural mesothelioma), immune system and cancer, immunotherapy, novel therapeutic approach

## Abstract

**Background:**

Malignant Pleural Mesothelioma (MPM) is an aggressive cancer of the mesothelial lining associated with exposure to airborne non-degradable asbestos fibers. Its poor response to currently available treatments prompted us to explore the biological mechanisms involved in its progression. MPM is characterized by chronic non-resolving inflammation; in this study we investigated which inflammatory mediators are mostly expressed in biological tumor samples from MPM patients, with a focus on inflammatory cytokines, chemokines and matrix components.

**Methods:**

Expression and quantification of Osteopontin (OPN) was detected in tumor and plasma samples of MPM patients by mRNA, immunohistochemistry and ELISA. The functional role of OPN was investigated in mouse MPM cell lines *in vivo* using an orthotopic syngeneic mouse model.

**Results:**

In patients with MPM, the protein OPN was significantly more expressed in tumors than in normal pleural tissues and predominantly produced by mesothelioma cells; plasma levels were elevated in patients and associated with poor prognosis. However, modulation of OPN levels was not significantly different in a series of 18 MPM patients receiving immunotherapy with durvalumab alone or with pembrolizumab in combination with chemotherapy, some of whom achieved a partial clinical response. Two established murine mesothelioma cell lines: AB1 and AB22 of sarcomatoid and epithelioid histology, respectively, spontaneously produced high levels of OPN. Silencing of the OPN gene (*Spp1*) dramatically inhibited tumor growth *in vivo* in an orthotopic model, indicating that OPN has an important promoting role in the proliferation of MPM cells. Treatment of mice with anti-CD44 mAb, blocking a major OPN receptor, significantly reduced tumor growth *in vivo*.

**Conclusion:**

These results demonstrate that OPN is an endogenous growth factor for mesothelial cells and inhibition of its signaling may be helpful to restrain tumor progression *in vivo*. These findings have translational potential to improve the therapeutic response of human MPM.

## Introduction

Malignant Pleural Mesothelioma (MPM) is an aggressive cancer of the mesothelial lining that covers the lungs. It is characterized by a non-resolving, long-lasting inflammation, driven by the presence of non-degradable asbestos fibers inhaled from the environment. Although asbestos production has been discontinued in several western countries in the ‘90s, MPM incidence is still rising, as the latency period for its development is very long (up to 20-40 years) and the peak is estimated around 2025-2030 (1–3). MPM is usually identified at advanced stages because there are no useful biomarkers for an early diagnosis, and radiological diagnostic tools are not effective for its early detection. This cancer has a very poor prognosis with a median survival time from presentation of approximately 9–12 months (2–4). MPM has been classified into three different histotypes: the most common type is the epithelioid (70%), the sarcomatoid (∼20%) has the worst prognosis, and an intermediate third histotype, the biphasic, is characterized by a combination of cells with both epithelioid and sarcomatoid morphology (2, 3).

Chronic inflammation triggered by the non-degradable asbestos fibers has been established as the first pathogenic step in the long chain of events that drives the development of MPM. Over several years, chronic inflammation causes DNA damage and accumulation of DNA mutations. Genetic abnormalities have been extensively studied in MPM; a wide range of different mutations was found in several genes, most prominently in the BRCA1-associated protein–1 (BAP1) gene, and in other genes: CDKN2A, Wnt, p16, TP53, SMACB1, NF2, PI3K (5–11). Recently, point mutations or overexpression of KRAS have been reported in a proportion of human MPM (12).

Malignant mesothelioma is a tumor dramatically resistant to chemotherapy. Despite the introduction of modern therapeutic interventions, only modest changes in survival have been observed over time (2–4, 13–16). Immunotherapy based on checkpoint blockade (ICB) is currently under investigation in clinical trials with - so far - disappointing results (17). Recently, clinical studies using a combination of nivolumab plus ipilimumab have reported a significant extension of patient survival, restricted to the sarcomatoid histotype (18). Another treatment modality that gained credit is the use of alternating electric fields, a noninvasive therapeutic approach that can complement chemotherapy in mesothelioma patients. A combination of cisplatin-based therapies with Tumor-Treating Fields (TTF) has shown *in vitro* anti-tumor activity (19) and clinical activity in a phase 2 study (20). Despite these encouraging successes, it is clear that more effective treatments are urgently needed to assist these patients, and for this we need to increase our knowledge on the biology of MPM, especially on the molecular pathways that govern its continuous proliferation and resistance to treatments.

Our group has a long-lasting interest in the mechanisms of the inflammatory cascade that actively support neoplastic transformation (tumor-promoting inflammation), a condition paradigmatically represented in malignant mesothelioma (21–25). In this study, we performed a transcriptomic analysis of genes of the inflammatory response in human mesothelioma tumor samples to identify which molecular pathways are mostly upregulated. Our attention was caught by the high expression of the *Spp1* gene, coding for the protein osteopontin (OPN). OPN is a highly phosphorylated matricellular protein produced by several cell types: macrophages, stromal and epithelial cells. OPN can interact with integrins and with the CD44 receptor and regulates several cell functional pathways, including cell motility, immune responses, cell proliferation and apoptosis (26–28). Furthermore, OPN is abundantly present in inflamed tissues favoring immune cell accumulation, retention of macrophages and activation of cell survival, thus exacerbating the chronic inflammatory response (29, 30). The expression of OPN in MPM is well known: several studies have investigated this protein as a potential diagnostic or prognostic biomarker (31–39); its functional role in malignant mesothelioma, however, has not been elucidated.

In this study, we have done a comprehensive analysis of the expression of OPN in human MPM patients, including patients undergoing checkpoint blockade immunotherapy, and *in vivo* studies using a murine orthotopic model of mesothelioma. Our findings demonstrate that OPN has an important functional role and promotes the progression of malignant mesothelioma.

## Materials and methods

### Mesothelioma patients

Tumor and plasma samples were obtained from patients with pathologically confirmed malignant mesothelioma admitted at the IRCCS Humanitas Clinical and Research Center (Rozzano, Milano-Italy). Samples were collected upon the signing of an informed consent and immediately frozen and stored in the Institutional Biobank. Plasma samples were obtained also from 18 MPM patients with epithelial histology treated with checkpoint blockade immunotherapy: 10 patients with durvalumab as single agent in second-line setting, 8 patients with pembrolizumab combined with carboplatin and pemetrexed in first-line setting. Plasma samples were collected also from 61 MPM patients enrolled in the ATREUS study (ClinicalTrials.gov, NCT02194231), a phase II, single arm, multicenter study aimed to explore the activity of trabectedin in second-line setting (40). Plasma samples were collected before start of therapy. All studies were conducted after approval by the Ethic Committee. Written informed consent was obtained from each patient before entering the study. Recommendations of the Declaration of Helsinki were followed.

The human mesothelioma cell lines CD288 and CD484 were derived from tumor samples of patients with diagnosed epithelioid MPM implanted in athymic nude mice, then established *in vitro*, as described (41).

### Murine mesothelioma cell lines

The murine mesothelioma cell lines AB1 (sarcomatoid histology) and AB22 (epithelioid histology), were generated in BALB/c mice upon intraperitoneal injection of crocidolite asbestos fibers and deposited in the Australian cell bank (42). Luciferase‐expressing AB1 and AB22 cells were kindly provided by Dr. M. Bianchi, San Raffaele Scientific Institute, Milan, Italy (43). Cell lines were cultured in RPMI 1640 (Lonza) supplemented with 10% FBS (Sigma), 2mM L-glutamine, 100 U/mL penicillin and 100 μg/mL streptomycin (Life Technologies Inc.) at 37°C and 5% CO2. To silence the Spp1 gene coding for osteopontin, AB1 and AB22 cells were stably transduced with the lentiviral vector MISSION shRNA (SHCLNG, 10041725MN, SIGMA). Viral particles were generated in HEK293T cells transfected with Lipofectamine2000 (ThermoFisher Scientific) according to manufacturer’s instruction. Selection of transduced cells was performed using Puromycin (2 ug/ml for three days after each defrosting). A non-targeting shRNA (scrambled) was used to transduce the control cell lines. All cell types were routinely checked for Mycoplasma contamination.

### *In vitro* colony assay

Proliferation of mesothelioma cell lines in the presence of anti-CD44 mAb or isotype control (BioXcell, BE0039, 5μg/ml) was quantified by staining with Crystal violet after 1 week; colonies were dissolved in pure DMSO and optical density measured by spectrophotomer at 590 nm.

### *In vivo* experiments in mice

Mice were used in compliance with national (D.L. N. 26, G.U. March 4, 2014) and international law and policies (EEC Council Directive 2010/63/EU, OJ L 276/33, 22-09-2010; National Institutes of Health Guide for the Care and Use of Laboratory Animals, (authorization N° 296/2020-PR), and US National Research Council, 2011). BALB/c mice 8 weeks-old were purchased from Charles River. The procedures for the syngeneic orthotopic mouse model have been previously described (44). AB1 and AB22 MPM cells were injected intra-thoracically. Mice were anesthetized with ketamine/xylazine and positioned on left lateral decubitus. The thoracic area was shaved and sterilized with 70% ethanol. An 8-10 mm skin incision was performed on the right thorax (close to the axillary cavity) and 5x10^4^ cells resuspended in 50 ul saline solution were injected between the third and the fourth costal space, with the needle perpendicularly oriented on the rib cage (29-gauge needle of a 500 ul syringe U100, BD Becton, Dickinson). In order to standardize the injection and avoid lung perforation, the needle was overmounted by a 200ul tip, properly cut to expose the needle of 3 mm only. After cell injection, mice were sutured and kept under a heating lamp to recover from the anesthesia. Tumor growth quantification was performed by *in vivo* imaging over time. Mice were i.p. injected with D-Luciferin (XenoLight D-Luciferin-K+ Salt, PerkinElmer; 150 mg Luciferin/kg body weight). Ten minutes after D-Luciferin injection, the bioluminescent signal was acquired using the IVIS Lumina III system (Perkin Elmer). During the acquisition procedure, mice were anesthetized with Isoflurane (XGI-8 Gas anesthesia system, Perkin Elmer). Data were analyzed with Living image 4.3.1 by designing a ROI on the thoracic area of each mouse. To block the CD44 receptor, mice were treated intra-peritoneally with anti-CD44 mAb (BioXcell, BE0039,10 mg/kg), or an irrelevant antibody at days 7, 12, 16, 19 post tumor injection, or otherwise specified in the figure legends.

### ELISA quantification of OPN

To quantify the production of human/murine OPN, cell supernatants or plasma samples were tested with commercial ELISA kits (R&D Systems), according to the manufacturer’s instructions. Data were analyzed with SoftMax Pro 5.3 software.

### Histopathology

Lungs and intra-thoracic masses of mice were fixed in 10% buffered formalin, routinely processed for histopathology, cut at 4 μm thickness, and stained with hematoxylin and eosin. Digital slides were obtained from haematoxylin and eosin-stained sections using the NanoZoomer S60 Digital slide scanner (Hamamatsu, C13210-01) and visualized by NDP.view2 Viewing software (Hamamatsu, U12388-01). For each case, pulmonary nodules were counted and subsequently manually outlined obtaining the area expressed in mm^2^.

### Immunohistochemistry

4-μm sections of paraffin-embedded human tissues were stained with primary antibodies anti-OPN (MAB14334, R&D System) or anti-CD206 (AF2534,R&D System). For murine tissues, 4-μm sections of paraffin-embedded lungs were stained as previously described (43). The primary antibodies used were anti-Iba1 (019-19741, Wako Chemicals), anti-CD206 (ab64693, Abcam), anti-CD3e (Sc-1127, Santa Cruz Biotechnology), anti-CD4 (4SM95; 14-9766-82, eBioscience), anti-CD8 (4SM15; 14-0808-82, eBioscience), anti OPN (MAB808, R&D System). Digital slides were obtained from immunostained sections by using the NanoZoomer S60 Digital slide scanner (Hamamatsu, C13210-01) and visualized by NDP.view2 Viewing software (Hamamatsu, U12388-01). For each case, 1 20X hot spot field was taken from the biggest 10 masses for every evaluated marker. Images were then processed in ImageJ software (http://rsb.info.nih.gov/ij/) to calculate the positive area/total area ratio expressed in percentage.

### Real-time RT-PCR

PureZOL RNA isolation reagent (BIORAD) was used to extract total RNA from tumor samples; cDNA was then synthesized from 2ug of total RNA with GeneAmp RNA PCR kit (applied Biosystems). Real-Time PCR was run using SYBR Green dye and 7900HT fast Real Time PCR system (Applied Biosystems). Primer Express Software (Applied Biosystems) was used to design the sequence of primer pairs specific for each gene (SIGMA). mRNA was normalized to GAPDH mRNA by subtracting the cycle threshold (Ct) value of GAPDH mRNA from the Ct value of the gene (ΔCt). ΔCt was then multiplied for an arbitrary unit (100 000). The sequences of primers are as follows:

hOPN Forward: 5’ AGTTTCGCAGACCTGACATCCAGT 3’
hOPN Reverse: 5’ TTCATAACTGTCCTTCCCACGGCT 3’
mOPN Forward: 5’ AGCCACAAGTTTCACAGCCACAAGG 3’
mOPN Reverse: 5’ TGAGAAATGAGCAGTTAGTATTCCTGC 3’

### TaqMan low density array

Four mesothelioma surgical samples and their corresponding normal tissues were used for low‐density array (LDA) analysis as previously described (44). The relative amount of each target gene mRNA to the mean of the five housekeeping genes (HPRT, 18S, GAPDH, B2M, and ACTB) was calculated as 2^–ΔCt^, where ΔCt = Ct – Ct_mean of housekeeping genes_. The fold‐change of each target gene mRNA to the corresponding normal tissue was calculated as 2^–ΔΔCt^, where ΔΔCt = ΔCt_target gene in tumor tissue_ – ΔCt_target gene in normal tissue_. The threshold cycle Ct was automatically given by the SDS2.2 software package (Applied Biosystems) (45).

### RNA seq analysis

Raw data were demultiplexed with bcl2fastq Conversion Software (Illumina). FastQC (46) was used for data quality check. Data analysis bcbio-nextgen (47) pipeline which was configured with hisat2 (48) as aligner using the Mus musculus *mm10* transcriptome and salmon (49) for gene counts assessment. DESeq2 (50) package was used for data post-processing and differential expression analysis. Counts were filtered retained only genes with at least 10 reads. shOPN cells were compared to control cells (CTR) to assess differentially expressed genes (DEGs) (p-adjust less than 0.05, multiple testing correction with False Discovery Rate). Enrichment analysis was used to associated genes with pathways using the *enrichPathway* function of clusterProfiler (51) R package using the Reactome database (52) mouse was set as organism, p-value cut-off was set to 0.05 and normalized gene counts were used as universe). Pheatmap (53) R package was used for DEGs visualization, clustering was done with the Ward method. Pathway barplot was done with the seaborn (54) package.

### Statistical analysis

Prism software (v8.0; GraphPad Software, San Diego, CA) was used to conduct appropriate statistical procedures, as specified in figure legends. Outliers were removed using the ROUT method. A p value < 0.05 was considered significant unless noted otherwise. Overall survival time was calculated from the date of surgery to the date of death or last contact. Statistical analyses of the results were performed using Unpaired t test with Welch’s correction.

## Results

### Osteopontin expression and plasma levels in malignant mesothelioma patients

**Figure 1:**
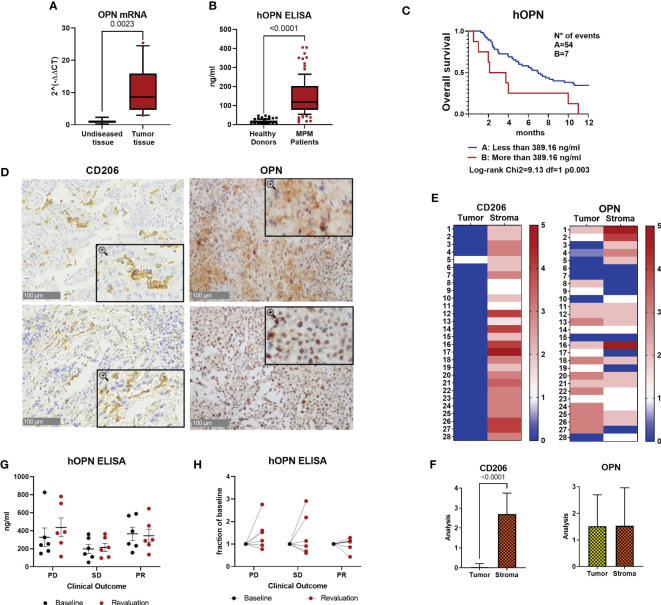
Osteopontin is overexpressed in human MPM patients. **(A)** Real Time PCR for the *Spp1* gene (osteopontin, OPN) in surgical human MPM samples. Comparison between tumor and undiseased adjacent tissues. Data are shown as mean ± SEM (Unpaired t test with Welch’s correction). **(B)** ELISA quantification of hOPN on plasma samples from 99 MPM patients and 101 healthy subjects. (ROUT, identify outlier and Unpaired t test with Welch’s correction). **(C)** Kaplan-Meier curves of overall survival according to OPN levels categorized based on CART analysis cut-off (n=61 MPM patients). **(D–F)** Representative images of immunohistochemistry in MPM tumor tissues stained for OPN or CD206 (40x, insert 100x) and semi-quantitative analysis in 28 cases (0=negative, 1 = 1-25% positivity, 2 = 26-50% positivity, 3= >50% positivity). Data are shown as mean ± SEM. (Unpaired t test with Welch’s correction). **(G, H)** ELISA quantification of hOPN on plasma samples from 18 MPM patients treated with immunotherapy. Blood was collected at baseline and after revalutation at 4-6 months. Patients with progressive disease (PD): 6 patients; stable disease (SD): 6 patients; partial response (PR): 6 patients.

To study the inflammatory environment of malignant pleural mesothelioma tissues, we performed a gene expression analysis using a TaqMan Low Density Array containing 91 genes related to the inflammatory response (45). RNA was extracted from 4 surgically resected tumor samples and from the adjacent un-diseased tissues. Several genes coding for cytokines/chemokines known to activate inflammatory cells (i.e., CCL2, CCL3, CCL7, CCL11, CCL20, CCL26, CXCL8 and CXCL1) were upregulated in MPM tissues, as well as the vascular growth factor VEGFα, PTGS2 coding for COX-2 and Spp1 coding for osteopontin (OPN) (**Supplementary Figure 1**). Spp1 results were confirmed in real-time PCR analysis performed on 15 MPM samples; mRNA levels were significantly higher in tumor tissues compared to un-diseased tissues (**Figure 1A**). OPN is a secreted matrix-related protein with multiple functions in healthy and pathological conditions (29). ELISA quantification in plasma was performed in MPM patients (n=99). OPN levels were significantly higher compared with healthy donors (n=101) (**Figure 1B**). In a series of 61 patients enrolled in a multicenter phase II study (ATREUS, ClinicalTrials.gov, NCT02194231) receiving the drug trabectedin as monotherapy (40), high plasma levels of OPN at baseline were significantly associated with worse overall survival (**Figure 1C**). To further characterize the expression of OPN in human MPM, we analyzed its immunoreactivity in 28 surgical human MPM tissues. Immunostaining for OPN was distinctly localized in the cytoplasm of tumor cells in 75% of the cases, while in other cases a diffuse staining was observed, in line with the secreted soluble form of this protein (**Figures 1D–F**). As macrophages are known producers of OPN, anti-CD206 immunostaining was also investigated; as expected, macrophage staining was selectively localized in the stroma and in some samples cytoplasmic staining for OPN was also detected in the stroma (**Figures 1D–F**). Next, we quantified the plasma levels of OPN in a series of 18 MPM patients receiving immunotherapy with Durvalumab alone or with Pembrolizumab in combination with chemotherapy. Baseline levels before treatment in patients with progressive disease (PD) did not differ from those of patients achieving a stable disease (SD) or a transient partial response (PR) (**Figure 1G**). Modulation of OPN levels after 4-6 months of therapy was similarly heterogeneous among patients; although the low numerosity does not allow to draw conclusions on this point, we noted that while 5/12 patients (PD+SD) showed increased levels compared to baseline values, none of the responding patients had increase of OPN levels at revaluation (**Figure 1H**).

Collectively, these results confirm the higher expression of OPN in tumor tissues and circulating blood of MPM patients compared to healthy donors and indicate that high OPN may be associated with unfavorable prognosis; however, OPN monitoring during ICB immunotherapy has not been useful to identify patients responding to treatment.

### Role of OPN in murine mesothelioma cell proliferation

**Figure 2:**
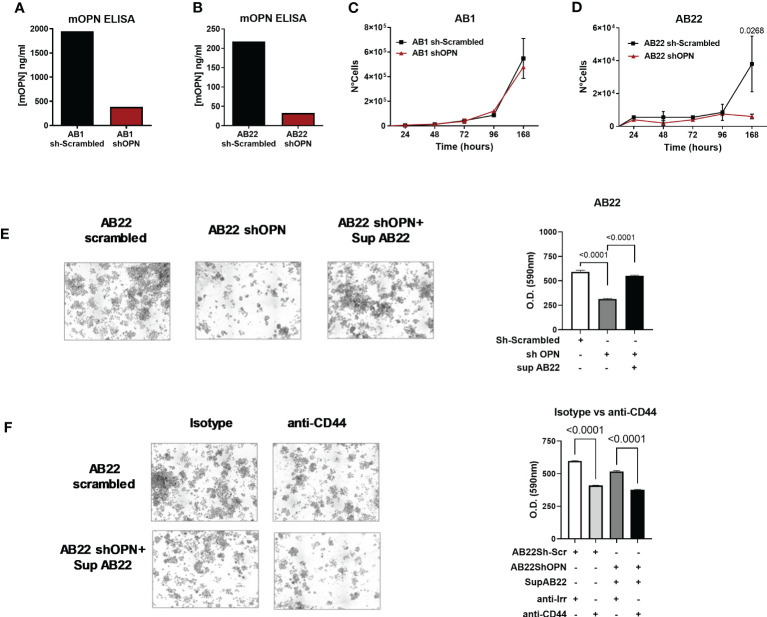
*In vitro* characterization of murine engineered MPM cell lines. **(A, B)** ELISA for mOPN on AB1 sh-scrambled and AB1 shOPN cells **(A)**, and AB22 sh-scrambled and AB22 shOPN cells **(B)**, showing the efficacy of silencing. **(C, D)** cell proliferation assay over time for AB1 **(C)** and AB22 **(D)** sh-scrambled and shOPN cells. Data are shown as mean ± SEM (Two-way ANOVA). **(E)** Representative images of the colony assay and quantification for AB22 scrambled cells, AB22 shOPN cells, also after addition of OPN-containing supernatant from scrambled cells. **(F)** images of the colony assay and quantification in the presence of a blocking anti-CD44 mAb (5 μg/ml). Blockade of CD44 inhibits cell proliferation in AB22 sh-scrambled cells and in AB22 shOPN cells exposed to OPN-containing supernatant. Data are shown as mean +/- SD (One-way ANOVA).

To test the functional activity of OPN we used two murine mesothelioma cell lines: AB1 cells and AB22 cells with sarcomatoid and epithelioid histology, respectively. Both cell lines spontaneously produced OPN, quantified by ELISA in cell supernatants. AB1 cells were high producers of OPN and secreted up to 1900 ng/ml (**Figure 2A**), while AB22 cells produced 220 ng/ml (**Figure 2B**). Using the lentivirus vector (MISSION shRNA) both AB1 and AB22 cell lines were successfully silenced for the Spp1 gene: 84% and 81%, respectively, (**Figures 2A, B**), though silencing was not complete in the AB1 cell line producing very high levels of OPN. The *in vitro* characterization of the engineered cells revealed that OPN silencing had no effect on the proliferation of AB1 cells, as AB1shOPN cells did not modify their growth behavior (**Figure 2C**). On the other hand, gene silencing dramatically reduced the proliferation ability in AB22 shOPN cells, compared with the scrambled-transduced cell line (AB22 sh-scrambled) (**Figure 2D**). In a colony assay, AB22 shOPN cells formed 47% less colonies than AB22 sh-scrambled cells (**Figure 2E**). To investigate if the addition of OPN restored their proliferation, silenced cells were treated with 30% conditioned medium from AB22 sh-scrambled cells: after 1 week, AB22 shOPN cells showed 1, 6 fold more colonies (**Figure 2E**).

To further confirm the involvement of OPN, we investigated the effect of blocking its major receptor CD44. Expression of CD44 by cancer cells was first checked by immunohistochemistry. Murine mesothelioma AB1 and AB22 cells stained strongly positive for CD44 **Supplementary Figure 2A**, in line with its ubiquitous nature (55). Likewise, two representative samples of human pleural mesothelioma expressed CD44 as shown in **Supplementary Figure 2B**. To block the receptor, AB22 cells were treated every other day with a blocking anti-CD44 mAb (5 μg/ml). Anti-CD44-treated cells had a significantly lower proliferation rate (**Figure 2F**); similar results were obtained also using AB22 shOPN cells that were exposed to the conditioned medium containing OPN (**Figure 2F**). Overall, these results indicate that OPN is an essential endogenous growth factor for the epithelioid AB22 cells. Furthermore, we tested two human MPM cell lines: CD288 and CD484; both cell lines spontaneously produce OPN (**Supplementary Figures 3A, B**). Also with human MPM cells, addition of anti-CD44 significantly decreased tumor cell proliferation (**Supplementary Figures 3C, D**).

**Figure 3:**
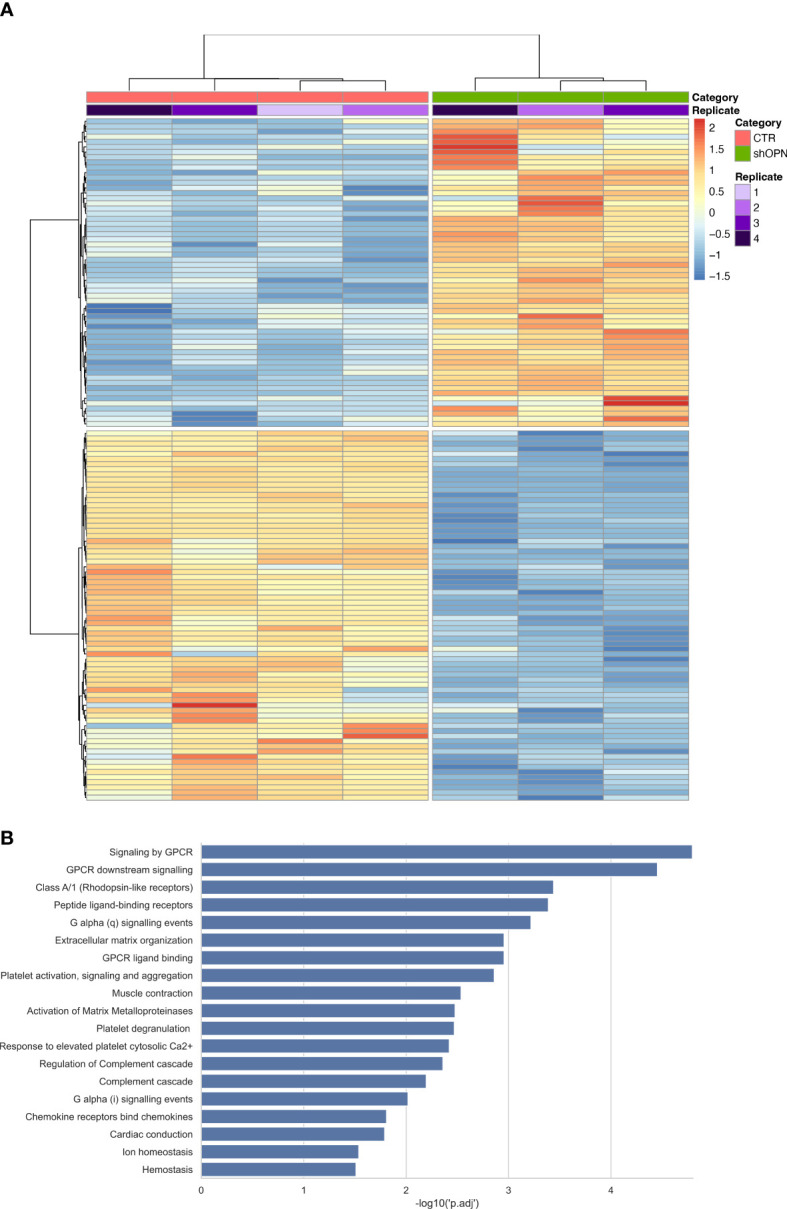
Transcriptome Sequencing analysis of AB22 sh-scrambled and AB22 shOPN cells. **(A)** The heatmap shows 132 deregulated gens (DEGs) from shOPN cells and comparison vs sh-scrambled cells (CTR). Supervised clustering shows the CTR samples with a red bar and shOPN samples with a green bar. Replicates are indicated with a violet scale color. Gene expression is shown with false color scale as indicated in the legend: red for positive values, blue for negative values. The darker the color, the higher the expression. **(B)** Pathway analysis software shows the pathways significantly associated with DEGs from the shOPN vs CTR comparison. Pathways are sorted from the most significant to the least, as indicated by the -log10 adjusted p-value on the x-axis.

A Transcriptome Sequencing (RNAseq) was performed on AB22 shOPN cells and results compared with AB22 sh-scrambled control cells. One hundred thirty-two differentially expressed genes (DEGs) were identified (**Figure 3A**). Reactome enrichment analysis confirmed that top DEGs were involved in biological processes such as immune system, cell proliferation and adhesion, molecular function regulator. The main enriched pathways were: peptide ligand-binding receptor Ga signaling, extracellular matrix organization, activation of MMPs and G-protein-coupled receptor (GPCR) signaling (involved in the downstream signaling of the receptor CD44) (**Figure 3B**). These findings indicate that loss of OPN has a relevant impact on fundamental biological processes of mesothelioma cells.

### Role of OPN in murine mesothelioma cell *in vivo* tumor growth

We next studied the *in vivo* growth of shOPN AB1 and AB22 engineered cells. As described by Digifico et al. (44), we set up an orthotopic model of murine mesothelioma that recapitulates the human MPM. In this model, direct intra-thorax injection of tumor cells was performed with a minimally invasive procedure. As confirmed by histological examinations, tumors developed along the pleura surface, further spreading and colonizing the most peripheral areas of the lungs, without forming any neoplastic mass outside the thoracic cavity. Importantly, acquisition over time of the bioluminescent signal from Luc-transduced cells was totally trustable as it perfectly correlated with the quantification of tumor areas detected with conventional histology (44).

**Figure 4:**
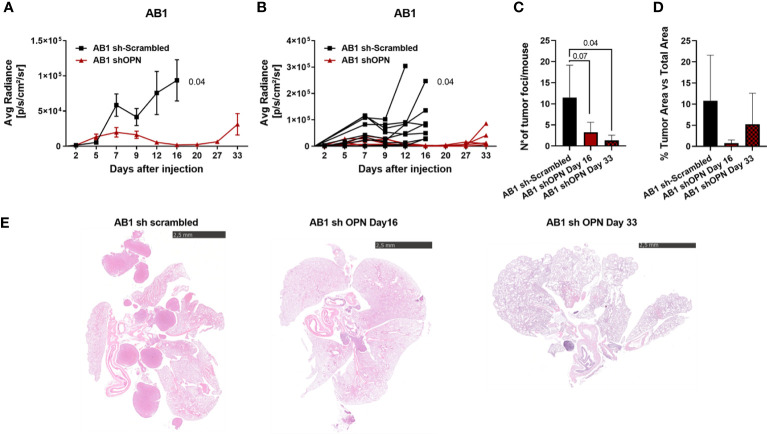
Silencing of OPN impairs the growth of AB1 cells *in vivo*. **(A, B)** *In vivo* growth of 5x10^4^ AB1 sh- scrambled or AB1 shOPN, injected intra-thoracically in BALB/c mice. **(A)** IVIS *in vivo* imaging luminescence signal, mean+/-SEM values of 9 mice sh-scrambled, 5 for shOPN; **(B)** luminescence signal values of each single mouse. **(C)** Histological quantification of tumor foci, and **(D)** of total tumor area. Data are shown as mean +/- SEM; (**A, B** Two-way ANOVA; **C, D**: One-way ANOVA). **(E)** Representative pictures of explanted lungs from tumor-bearing mice. AB1 sh-scrambled cells (left), AB1 shOPN cells at day 16 (middle) and AB1 shOPN cells at day 33 (right). Bars represent 2.5 mm.

shOPN Luc-expressing AB1 and AB22 cells and their scrambled controls (5x10^4^ cells) were injected intra-thoracically in syngeneic BALB/c mice and tumor growth was followed by IVIS Lumina III system up to the day of sacrifice. At day 14 post injection we observed that OPN silencing almost completely abrogated tumor growth *in vivo* of shOPN AB1 cells, as detected by IVIS signal, as well as by histological quantification of total tumor area (**Supplementary Figures 4A–C**). Immunostaining of explanted tumors evidenced the significantly reduced expression of OPN in silenced tumors (**Supplementary Figures 4D, E**). A longer experiment confirmed this finding of growth inhibition and revealed that shOPN AB1 cells started growing again by day 33, but only in 2/5 mice (**Figures 4A, B**). By histological examination, the number of tumor foci at day 33 was still significantly reduced in mice bearing OPN-silenced tumor cells (**Figures 4C–E**).

**Figure 5:**
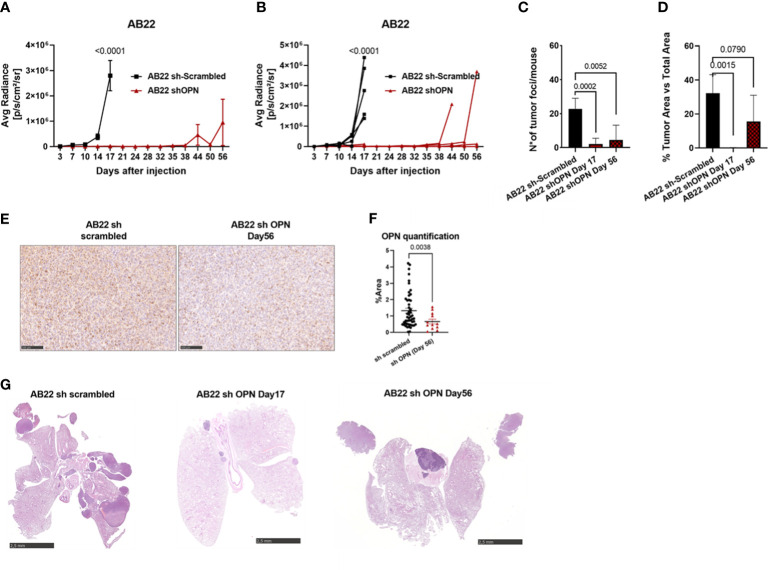
Silencing of OPN impairs the growth of AB22 cells *in vivo*. **(A, B)** *In vivo* growth of 5x10^4^ AB22 sh- scrambled or AB22 shOPN, injected intra-thoracically in BALB/c mice. **(A)** IVIS *in vivo* imaging luminescence signal, mean+/-SEM values of 5 mice for sh-scrambled, 5 for shOPN; **(B)** luminescence signal values of each single mouse. **(C)** Histological quantification of number of tumor foci, and **(D)** total tumor area. Data are shown as mean +/- SEM; (**A, B** Two-way ANOVA; **C, D**: One-way ANOVA; **E**: Unpaired t test with Welch’s correction). **E, F**) immunohistochemistry for OPN in explanted tumors and representative pictures, bars represent 100 µm. **(G)** Representative pictures of explanted lungs from mice bearing control AB22 sh-scrambled cells (left), AB22 shOPN cells at day 17 (middle) and AB22 shOPN cells at day 56 (right). Bars represent 2.5 mm.

With AB22 epithelioid cells, a first *in vivo* experiment demonstrated that OPN silencing strongly reduced tumor growth (**Supplementary Figures 5A–C**) and OPN expression in tumors (**Supplementary Figures 5D, E**). In a second *in vivo* experiment with longer time points, mice injected with scrambled cells had to be sacrificed at day 17, while endpoint for mice injected with silenced cells was at days 45-56 (**Figures 5A, B**). Quantification of tumor foci and tumor area was significantly reduced in shOPN cells at later times, only few masses were visible in 3/5 mice (**Figures 5C, D**). In the explanted tumors, expression of OPN detected by immunohistochemistry was indeed lower in silenced tumors (**Figures 5E, F**). **Figure 5G** shows representative pictures of tumor load around the lungs of mice injected with control AB22 cells or shOPN cells at different time points.

Taken together, these data indicate that OPN in both tumor histotypes is an essential growth factor supporting tumor progression *in vivo*.

### Inhibition of OPN signaling through the CD44 receptor reduces tumor growth *in vivo*

**Figure 6:**
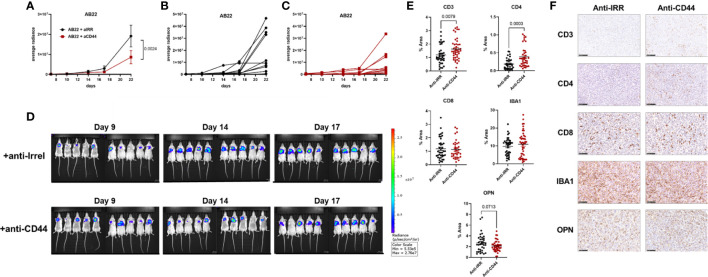
Treatment with blocking anti-CD44 mAbs impairs *in vivo* growth of murine mesothelioma cells. **(A–C)** Effect of anti-CD44 mAbs on AB22 tumor cell growth. Mice were treated intra-peritoneally with anti-CD44 (10 mg/kg) or with irrelevant mAbs at day (7, 12, 16, 19) post tumor injection. Data are expressed as average radiance, **(A)** mean+/-SEM values of 10 mice; **(B, C)** values of each single mouse; **(D)** images of IVIS acquisition of LUC signal at different time points. **(E, F)** Representative images of immunohistochemistry of explanted tumors and relative quantification, each dot represents a single ROI. Tumor slices were stained with mAbs against CD3, CD4, CD8, IBA1 (macrophages) and OPN; bars represent 100 µm. Data are shown as mean +/- SEM (Statistical analysis: **(A)**, Two-way ANOVA; **(E)** Unpaired t test with Welch’s correction).

Since our *in vivo* experiments revealed a clear role of OPN in promoting *in vivo* proliferation of murine MPM, experiments to block OPN were undertaken. A commercial aptamer, OPN-R3 (56, 57), able to specifically block OPN was first used. Repeated intraperitoneal injections did not affect tumor growth of AB22 cells (**Supplementary Figure 6**). We then turned to use blocking antibodies against CD44. Mice were treated with anti-CD44 mAb (10 mg/kg) at day 7, 12, 16, 19 post tumor implantation. With the AB1 cell line we did not observe a significant reduction of tumor growth over time (not shown); this finding is likely explained since AB1 cells are very high producer of OPN, secreting 10 times more OPN compared with AB22 cells (**Figures 2A, B**). We therefore tested the engineered shOPN AB1 cells, where production of OPN was not totally abrogated. Treatment of mice with anti-CD44 antibodies significantly reduced tumor growth of shOPN AB1 cells (**Supplementary Figure 7**). Next, the sh scrambled AB22 cell line was used for the same type of experiment; growth of AB22 cells (10 mice/group) was substantially reduced (p= 0.0024) in anti-CD44-treated mice compared with mice treated with the irrelevant antibody (**Figures 6A–D**). By immunohistochemistry, we observed a significantly higher number of CD3+ and CD4+ cells, and a trend to decreased expression of OPN (**Figures 6E, F**). Instead, the infiltration of CD8+ T cells and that of macrophages (IBA1+ cells) was not changed (**Figures 6E, F**).

Overall, these data demonstrate that OPN produced by mesothelioma cells sustains the proliferation of cancer cells, and that inhibition of OPN signaling significantly reduces the pro-tumoral effects of OPN on the progression of malignant mesothelioma.

## Discussion

In this paper we studied the expression of OPN in tumor and plasma samples of MPM patients and performed functional studies with murine mesothelioma cell lines using an orthotopic mouse model. Considerable experimental evidence indicates that OPN expression is enhanced in a variety of pathological processes such as chronic inflammation, autoimmune diseases and cancer (26–29, 58, 59). Various studies reported that elevated levels of OPN are detected in different types of malignancies: breast, prostate, colorectal and lung cancer, melanoma and hepatic carcinoma. Most studies agree that OPN plays a key role in cancer progression by enhancing proliferation, motility and invasion of tumor cells and the process of angiogenesis (60–67). These tumor-promoting functions are achieved via different mechanisms: binding to integrins or CD44 receptor increases the integrin-stimulated FAK-Src-Rho pathway, cancer cell adhesion and survival, while activation of MMPs and matrix remodeling enhances tumor cell invasiveness; PI3K/Akt activation promotes tumor angiogenesis, recruitment of endothelial cells and tumor growth (60, 68).

In malignant mesothelioma, OPN has been extensively studied as diagnostic biomarker, frequently in association with another molecule: mesothelin. Using plasma or serum samples from MPM patients, detection of OPN levels by ELISA was found higher in patients in comparison with healthy donors, and even with healthy individuals exposed to asbestos (32, 35, 69). OPN has been investigated also as prognostic biomarker of treatment outcome: elevated OPN levels have been associated with an unfavorable prognosis in a number of studies (35–38, 70).

However, the real clinical utility of OPN as early diagnostic marker has also been questioned, due to its low sensitivity and specificity; for instance, circulating levels of OPN did not discriminate between chronic inflammatory and malignant lung diseases (71, 72).

In this paper we found that OPN in MPM patients is highly expressed both as mRNA and protein in tumor tissues, and as ELISA levels in the peripheral blood. Immunohistochemistry for OPN shows both a cytoplasmic staining in tumor cells as well as a diffused staining, in line with its secreted form. Analysis of the stroma with the macrophage marker CD206 indicates that in some cases macrophages also produce OPN, as already known in the literature (73–75). In a cohort of MPM patients enrolled in the ATREUS study (ClinicalTrials.gov, NCT02194231) (40), those patients with high baseline OPN levels indeed had a lower overall survival. On the other hand, modulation of OPN levels was not significantly different in patients receiving ICB immunotherapy, some of whom achieved a transient partial response. These results are in line with the widespread opinion that OPN is not a robust diagnostic or prognostic biomarker of disease for MPM (4, 71, 72)

While studies on the functional role of OPN in several types of tumors are available (58–65), its biological effects in malignant mesothelioma have not been clarified. To shed light on the functional activities of OPN in this neoplasia, we used two MPM mouse cell lines: AB22 of epithelioid phenotype and AB1 with sarcomatoid phenotype. Both spontaneously produced OPN, the latter up to large amounts. Silencing of OPN caused a strong delay in the proliferation of AB22 *in vitro*. Notably, the addition of cell supernatant containing OPN stimulated proliferation again and this OPN-induced proliferation was substantially reduced by blocking anti-CD44 mAbs. These results confirmed, *in vitro*, the important role of the axis OPN-CD44 in the proliferative expansion of mesothelioma cells. Silenced cells were compared with control cells in a Transcriptome Sequencing; the analysis revealed that the top deregulated genes are involved in receptor signaling pathways, primarily GPCR signaling, chemokines and migration, as well as matrix regulation. Of note, engagement of the receptor CD44 involves downstream signaling via G-protein-coupled receptors, in addition to other signaling pathways (76, 77). These results indicate that loss of OPN impacts on biologically important functions of this molecules through its receptors. 

*In vivo* experiments using a recently optimized orthotopic mouse model of mesothelioma clearly indicated that loss of OPN strongly reduced the proliferation of MPM cells, even in the case of AB1 cells where the gene was only partially switched off. In longer experiments few tumors started to grow again, only in some mice. Macrophages are known to produce OPN, however, silencing in cancer cells was sufficient to give a strong retardation of tumor growth. This finding indicates the importance of OPN as a cell autonomous growth factor for mesothelioma cells, apparently more relevant than the host derived OPN.

Subsequent experiments aimed to provide a proof of principle that pharmacological inhibition of OPN signaling, by targeting the receptor CD44, could indeed decrease mesothelioma cell growth *in vivo*. We found that administration to mice of a blocking anti-CD44 mAb, significantly reduced tumor proliferation of AB22 naïve cells as well as that of shOPN AB1 cells. This finding is remarkable because of the redundancy of receptors used by OPN. This molecule, in fact, binds several integrins in addition to CD44, but our results demonstrate specific inhibition of CD44 was sufficient to have positive therapeutic effects in mice. CD44 receptor has long been considered as a potential therapeutic target in cancer, as it initiates and modulates several signaling networks that are important in tumor progression, metastasis and chemoresistance (78). Being a pleiotropic receptor expressed in multiple tissues, it would not seem a good target for therapeutic purpose. However, CD44 is upregulated in a variety of cancers and alternatively spliced variant isoforms (e.g. CD44v6) are mostly expressed in tumors, particularly in advanced stages. Furthermore, CD44 expression has been associated to the process of Epithelial to Mesenchymal Transition and is a typical receptor of cancer stem cells (79). A number of studies validated the potential of CD44 as a therapeutic target in various tumor types (78). In malignant mesothelioma the expression of CD44, alone or in association with other molecules, has been mainly investigated as marker of disease, not for therapeutic potential (80–83). Our *in vitro* and *in vivo* results suggest that inhibition of OPN signaling might be a possible strategy to restrain mesothelioma cell growth.

Of interest, it has been recently reported that OPN is able to bind to another molecule, the ligand of the Inducible T-cell costimulatory (ICOS-L) (84, 85). ICOS-L, a B7 family member, sustains T cell immunity and the antitumor response by binding to ICOS, a costimulatory receptor expressed on activated T cells (86). Binding of ICOS-L to OPN, instead promoted tumor metastases in a mouse breast cancer model (85). ICOS-L is expressed also in human malignant mesothelioma (87). Thus, also for this new molecular partner of OPN, interfering with this binding may be explored as a new therapeutic approach.

In conclusion, on the basis of the experimental evidence obtained in this study, our working hypothesis that OPN represents an essential endogenous growth factor for mesothelioma cells, with a relevant role in driving tumor cell survival and proliferation, is confirmed. These results increase our knowledge on the biology of mesothelioma and suggest that therapeutic strategies based on OPN inhibition could have an impact on the management and survival of patients with MPM.

## Data availability statement

The original contributions presented in the study are publicly available. This data can be found here: https://www.ebi.ac.uk/ena/ under the accession number PRJEB58309.

## Ethics statement

The studies involving human participants were reviewed and approved by Ethical Committee of IRCCS Humanitas Research Institute. The patients/participants provided their written informed consent to participate in this study. The animal study was reviewed and approved by Ministry of Health.

## Author contributions

ED, ME, AU, CA, LB, and CB contributed to study design, performed all the experiments, analyzed and interpreted the data. LM and SM performed the experiments of RNAseq. LB, CR, PB, and MR supervised the pathological examinations. PZ and DP took care of patient enrollement, treatment and sample collection. Writing-review & editing: CB, MD’I, and PA. All authors contributed to the article and approved the submitted version.

## Supplementary material

Supplementary Figure 1 Transcriptomic analysis of inflammatory genes in human malignant mesothelioma. Gene expression profiling of four surgical human MPMs samples using TaqMan Low Density Array containing inflammatory 91 genes. Data are shown as fold increase in tumor samples relative to the non-involved pleural tissue from each paired patient. Selected genes are shown for which at least 2 samples showed upregulation over normal tissues.

Supplementary Figure 2 Immunohistochemistry of CD44 expression by murine and human mesothelioma. a) murine mesothelioma tumors (AB1 and AB22) grown *in vivo* in mice. b) MPM1 and MPM2 are human mesothelioma surgical samples.

Supplementary Figure 3
*In vitro* characterization of hMPM cell lines. **(A, B)** ELISA for hOPN spontaneously produced by the cell lines CD288 and CD484 (epithelioid phenotype). **(C, D)** Representative images of colony assays and relative quantification: addition of a blocking anti-CD44 mAb (5 mg/ml) inhibits cell proliferation. Data are shown as mean +/- SD (One-way ANOVA).

Supplementary Figure 4 Silencing of OPN impairs the growth of murine AB1 mesothelioma cells *in vivo*. a-b-c) *In vivo* growth of 5x10^4^ AB1 sh-scrambled or AB1 shOPN, injected intra-thoracically in BALB/c mice. **(A)** IVIS *in vivo* imaging luminescence signal, mean+/-SEM values of 5 mice; **(B)** Histological quantification of total tumor area. **(C)** Representative images of IVIS acquisition of LUC signal at different time points. **(D, E)** Immunohistochemistry of explanted tumors, relative quantification for the staining of OPN and representative pictures. Data are shown as mean +/- SEM (**A** Two-way ANOVA; **B, D**: Unpaired t-test with Welsh correction).

Supplementary Figure 5 Silencing of OPN impairs the growth of murine AB22 mesothelioma cells *in vivo*. **(A–C)** *In vivo* growth of 5x10^4^ AB22 sh-scrambled or AB22 shOPN, injected intra-thoracically in BALB/c mice. **(A)** IVIS *in vivo* imaging luminescence signal, mean+/-SEM values of 5 mice; **(B)** Histological quantification of total tumor area. **(C)** Representative images of IVIS acquisition of LUC signal at different time points. **(D, E)** Immunohistochemistry of explanted tumors, relative quantification for the staining of OPN and representative pictures. Data are shown as mean +/- SEM (**A** Two-way ANOVA; **B**, **D**: Unpaired t-test with Welsh correction).

Supplementary Figure 6 Inhibition of OPN with the aptamer OPN-R3 does not affect the *in vivo* growth of murine AB22 mesothelioma cells. Results of IVIS *in vivo* imaging luminescence signal, mean+/-SEM values of 5 mice per group.

Supplementary Figure 7 Treatment with blocking anti-CD44 mAbs impairs *in vivo* growth of murine mesothelioma cells. **(A, C)** Effect of anti-CD44 mAbs on AB1 shOPN tumor growth. Mice were treated intra-peritoneally with anti-CD44 (10 mg/kg) or with irrelevant mAbs at day (4, 7, 12, 16, 19) post tumor injection. Data are expressed as average radiance, **(A)** mean+/-SEM values of 5 mice; **(B)** values of single mice; **(C)** Representative images of IVIS acquisition of LUC signal at different time points.

Supplementary Table 1 Single-Cell Transcriptome Sequencing analysis: list of DEGs.

Supplementary Table 2 Single-Cell Transcriptome Sequencing analysis: list of pathways.

## References

[1] Tsao AS Wistuba I Roth JA Kindler HL. Malignant pleural mesothelioma. J Clin Oncol (2009) 27(12):2081–90. doi: 10.1200/JCO.2008.19.8523 PMC4881753 19255316

[2] Yap TA Aerts JG Popat S Fennell DA. Novel insights into mesothelioma biology and implications for therapy. Nat Rev Cancer (2017) 17(8):475–88. doi: 10.1038/nrc.2017.42 28740119

[3] Carbone M Adusumilli PS Alexander HR Baas P Bardelli F Bononi A. Mesothelioma: scientific clues for prevention, diagnosis, and therapy. CA Cancer J Clin (2019) 69(5):402–29. doi: 10.3322/caac.21572 PMC8192079 31283845

[4] Janes SM Alrifai D Fennell DA. Perspectives on the treatment of malignant pleural mesothelioma. N Engl J Med (2021) 385(13):1207–18. doi: 10.1056/NEJMra1912719 34551230

[5] Bott M Brevet M Taylor BS Shimizu S Ito T Wang L. The nuclear deubiquitinase BAP1 is commonly inactivated by somatic mutations and 3p21.1 losses in malignant pleural mesothelioma. Nat Genet (2011) 43(7):668–72. doi: 10.1038/ng.855 PMC4643098 21642991

[6] Bruno F Baratti D Martinetti A Morelli D Sottotetti E Bonini C. Mesothelin and osteopontin as circulating markers of diffuse malignant peritoneal mesothelioma: a preliminary study. Eur J Surg Oncol (2018) 44(6):792–8. doi: 10.1016/j.ejso.2018.02.010 29503128

[7] Bueno R Stawiski EW Goldstein LD Durinck S De Rienzo A Modrusan Z. Comprehensive genomic analysis of malignant pleural mesothelioma identifies recurrent mutations, gene fusions and splicing alterations. Nat Genet (2016) 48(4):407–16. doi: 10.1038/ng.3520 26928227

[8] Brich S Bozzi F Perrone F Tamborini E Cabras AD Deraco M. Fluorescence *in situ* hybridization (FISH) provides estimates of minute and interstitial BAP1, CDKN2A, and NF2 gene deletions in peritoneal mesothelioma. Mod Pathol (2020) 33(2):217–27. doi: 10.1038/s41379-019-0371-0 31570769

[9] Sculco M La Vecchia M Aspesi A Pinton G Clavenna MG Casalone E. Malignant pleural mesothelioma: germline variants in DNA repair genes may steer tailored treatment. Eur J Cancer Oxf Engl 1990 (2022) 163:44–54. doi: 10.1016/j.ejca.2021.12.023 35032816

[10] Testa JR Cheung M Pei J Below JE Tan Y Sementino E. Germline BAP1 mutations predispose to malignant mesothelioma. Nat Genet (2011) 43(10):1022–5. doi: 10.1038/ng.912 PMC3184199 21874000

[11] Yoshikawa Y Emi M Hashimoto-Tamaoki T Ohmuraya M Sato A Tsujimura T. High-density array-CGH with targeted NGS unmask multiple noncontiguous minute deletions on chromosome 3p21 in mesothelioma. Proc Natl Acad Sci U S A (2016) 113(47):13432–7. doi: 10.1073/pnas.1612074113 PMC5127333 27834213

[12] Marazioti A Krontira AC Behrend SJ Giotopoulou GA Ntaliarda G Blanquart C. KRAS signaling in malignant pleural mesothelioma. EMBO Mol Med (2022) 14(2):e13631. doi: 10.15252/emmm.202013631 34898002 PMC8819314

[13] Zucali PA De Vincenzo F Perrino M Digiacomo N Cordua N D’Antonio F. Advances in drug treatments for mesothelioma. Expert Opin Pharmacother (2022) 23(8):929–46. doi: 10.1080/14656566.2022.2072211 35508368

[14] Tsao AS Pass HI Rimner A Mansfield AS. New era for malignant pleural mesothelioma: updates on therapeutic options. J Clin Oncol (2022) 40(6):681–92. doi: 10.1200/JCO.21.01567 PMC8853621 34985934

[15] Kindler HL Novello S Bearz A Ceresoli GL Aerts JGJV Spicer J. Anetumab ravtansine versus vinorelbine in patients with relapsed, mesothelin-positive malignant pleural mesothelioma (ARCS-m): a randomised, open-label phase 2 trial. Lancet Oncol (2022) 23(4):540–52. doi: 10.1016/S1470-2045(22)00061-4 PMC10512125 35358455

[16] Nicolini F Bocchini M Bronte G Delmonte A Guidoboni M Crinò L. Malignant pleural mesothelioma: state-of-the-Art on current therapies and promises for the future. Front Oncol (2019) 9:1519. doi: 10.3389/fonc.2019.01519 32039010 PMC6992646

[17] Tagliamento M Bironzo P Curcio H De Luca E Pignataro D Rapetti SG. A systematic review and meta-analysis of trials assessing PD-1/PD-L1 immune checkpoint inhibitors activity in pre-treated advanced stage malignant mesothelioma. Crit Rev Oncol Hematol (2022) 172:103639. doi: 10.1016/j.critrevonc.2022.103639 35192932

[18] Baas P Scherpereel A Nowak AK Fujimoto N Peters S Tsao AS. First-line nivolumab plus ipilimumab in unresectable malignant pleural mesothelioma (CheckMate 743): a multicentre, randomised, open-label, phase 3 trial. Lancet Lond Engl (2021) 397(10272):375–86. doi: 10.1016/S0140-6736(20)32714-8 33485464

[19] Mannarino L Mirimao F Panini N Paracchini L Marchini S Beltrame L. Tumor treating fields affect mesothelioma cell proliferation by exerting histotype-dependent cell cycle checkpoint activations and transcriptional modulations. Cell Death Dis (2022) 13(7):612. doi: 10.1038/s41419-022-05073-4 35840560 PMC9287343

[20] Ceresoli GL Aerts JG Dziadziuszko R Ramlau R Cedres S van Meerbeeck JP. Tumour treating fields in combination with pemetrexed and cisplatin or carboplatin as first-line treatment for unresectable malignant pleural mesothelioma (STELLAR): a multicentre, single-arm phase 2 trial. Lancet Oncol (2019) 20(12):1702–9. doi: 10.1016/S1470-2045(19)30532-7 31628016

[21] Digifico E Belgiovine C Mantovani A Allavena P. Microenvironment and immunology of the human pleural malignant mesothelioma. In: Ceresoli GL Bombardieri E D’Incalci M, editors. Mesothelioma: from research to clinical practice. Cham: Springer International Publishing (2019). p. 69–84. doi: 10.1007/978-3-030-16884-1_5

[22] Mantovani A Allavena P Marchesi F Garlanda C. Macrophages as tools and targets in cancer therapy. Nat Rev Drug Discovery (2022) 21(11):799–820. doi: 10.1038/s41573-022-00520-5 35974096 PMC9380983

[23] Germano G Frapolli R Belgiovine C Anselmo A Pesce S Liguori M. Role of macrophage targeting in the antitumor activity of trabectedin. Cancer Cell (2013) 23(2):249–62. doi: 10.1016/j.ccr.2013.01.008 23410977

[24] Liguori M Digifico E Vacchini A Avigni R Colombo FS Borroni EM. The soluble glycoprotein NMB (GPNMB) produced by macrophages induces cancer stemness and metastasis via CD44 and IL-33. Cell Mol Immunol (2021) 18(3):711–22. doi: 10.1038/s41423-020-0501-0 PMC8027814 32728200

[25] Mantovani A Marchesi F Malesci A Laghi L Allavena P. Tumour-associated macrophages as treatment targets in oncology. Nat Rev Clin Oncol (2017) 14(7):399–416. doi: 10.1038/nrclinonc.2016.217 28117416 PMC5480600

[26] Hattori T Iwasaki-Hozumi H Bai G Chagan-Yasutan H Shete A Telan EF. Both full-length and protease-cleaved products of osteopontin are elevated in infectious diseases. Biomed (2021) 9(8):1006. doi: 10.3390/biomedicines9081006 PMC8394573 34440210

[27] O’Regan AW Nau GJ Chupp GL Berman JS. Osteopontin (Eta-1) in cell-mediated immunity: teaching an old dog new tricks. Immunol Today (2000) 21(10):475–8. doi: 10.1016/S0167-5699(00)01715-1 11071524

[28] Scatena M Liaw L Giachelli CM. Osteopontin: a multifunctional molecule regulating chronic inflammation and vascular disease. Arterioscler Thromb Vasc Biol (2007) 27(11):2302–9. doi: 10.1161/ATVBAHA.107.144824 17717292

[29] Denhardt DT Noda M O’Regan AW Pavlin D Berman JS. Osteopontin as a means to cope with environmental insults: regulation of inflammation, tissue remodeling, and cell survival. J Clin Invest (2001) 107(9):1055–61. doi: 10.1172/JCI12980 PMC209291 11342566

[30] Lund SA Giachelli CM Scatena M. The role of osteopontin in inflammatory processes. J Cell Commun Signal (2009) 3(3–4):311–22. doi: 10.1007/s12079-009-0068-0 PMC2778587 19798593

[31] Schillebeeckx E van Meerbeeck JP Lamote K. Clinical utility of diagnostic biomarkers in malignant pleural mesothelioma: a systematic review and meta-analysis. Eur Respir Rev Off J Eur Respir Soc (2021) 30(162):210057. doi: 10.1183/16000617.0057-2021 PMC9489015 34789461

[32] Pass HI Lott D Lonardo F Harbut M Liu Z Tang N. Asbestos exposure, pleural mesothelioma, and serum osteopontin levels. N Engl J Med (2005) 353(15):1564–73. doi: 10.1056/NEJMoa051185 16221779

[33] Chen Z Gaudino G Pass HI Carbone M Yang H. Diagnostic and prognostic biomarkers for malignant mesothelioma: an update. Transl Lung Cancer Res (2017) 6(3):259–69. doi: 10.21037/tlcr.2017.05.06 PMC5504120 28713671

[34] Ahmed M Behera R Chakraborty G Jain S Kumar V Sharma P. Osteopontin: a potentially important therapeutic target in cancer. Expert Opin Ther Targets (2011) 15(9):1113–26. doi: 10.1517/14728222.2011.594438 21718227

[35] Grigoriu BD Scherpereel A Devos P Chahine B Letourneux M Lebailly P. Utility of osteopontin and serum mesothelin in malignant pleural mesothelioma diagnosis and prognosis assessment. Clin Cancer Res (2007) 13(10):2928–35. doi: 10.1158/1078-0432.CCR-06-2144 17504993

[36] Hollevoet K Nackaerts K Gosselin R De Wever W Bosquée L De Vuyst P. Soluble mesothelin, megakaryocyte potentiating factor, and osteopontin as markers of patient response and outcome in mesothelioma. J Thorac Oncol (2011) 6(11):1930–7. doi: 10.1097/JTO.0b013e3182272294 21841505

[37] Arnold DT De Fonseka D Hamilton FW Rahman NM Maskell NA. Prognostication and monitoring of mesothelioma using biomarkers: a systematic review. Br J Cancer (2017) 116(6):731–41. doi: 10.1038/bjc.2017.22 PMC5355927 28170372

[38] Pass HI Alimi M Carbone M Yang H Goparaju CM. Mesothelioma biomarkers: a review highlighting contributions from the early detection research network. Cancer Epidemiol biomark Prev (2020) 29(12):2524–40. doi: 10.1158/1055-9965.EPI-20-0083 32699075

[39] Katz SI Roshkovan L Berger I Friedberg JS Alley EW Simone CB. Serum soluble mesothelin-related protein (SMRP) and fibulin-3 levels correlate with baseline malignant pleural mesothelioma (MPM) tumor volumes but are not useful as biomarkers of response in an immunotherapy trial. Lung Cancer Amst Neth (2021) 154:5–12. doi: 10.1016/j.lungcan.2021.01.011 33561782

[40] Cortinovis D Grosso F Carlucci L Zucali PA Pasello G Tiseo M. Trabectedin in malignant pleural mesothelioma: results from the multicentre, single arm, phase II ATREUS study. Clin Lung Cancer (2021) 22(4):361–70. doi: 10.1016/j.cllc.2020.06.028 32732073

[41] Vázquez R Licandro SA Astorgues-Xerri L Lettera E Panini N Romano M. Promising *in vivo* efficacy of the BET bromodomain inhibitor OTX015/MK-8628 in malignant pleural mesothelioma xenografts. Int J Cancer (2017) 140(1):197–207. doi: 10.1002/ijc.30412 27594045

[42] Davis MR Manning LS Whitaker D Garlepp MJ Robinson BW. Establishment of a murine model of malignant mesothelioma. Int J Cancer (1992) 52(6):881–6. doi: 10.1002/ijc.2910520609 1459729

[43] Mezzapelle R Rrapaj E Gatti E Ceriotti C Marchis FD Preti A. Human malignant mesothelioma is recapitulated in immunocompetent BALB/c mice injected with murine AB cells. Sci Rep (2016) 6:22850. doi: 10.1038/srep22850 26961782 PMC4785401

[44] Digifico E Erreni M Colombo FS Recordati C Migliore R Frapolli R. Optimization of a luciferase-expressing non-invasive intrapleural model of malignant mesothelioma in immunocompetent mice. Cancers (2020) 12(8):2136. doi: 10.3390/cancers12082136 32752156 PMC7465989

[45] Erreni M Bianchi P Laghi L Mirolo M Fabbri M Locati M. Expression of chemokines and chemokine receptors in human colon cancer. Methods Enzymol (2009) 460:105–21. doi: 10.1016/S0076-6879(09)05205-7 19446722

[46] Babraham Bioinformatics. FastQC a quality control tool for high throughput sequence data. Available at: https://www.bioinformatics.babraham.ac.uk/projects/fastqc/.

[47] Contents [[/amp]]mdash; bcbio-nextgen 1.2.9 documentation. Available at: https://bcbio-nextgen.readthedocs.io/en/latest/.

[48] Pertea M Kim D Pertea GM Leek JT Salzberg SL. Transcript-level expression analysis of RNA-seq experiments with HISAT, StringTie and ballgown. Nat Protoc (2016) 11(9):1650–67. doi: 10.1038/nprot.2016.095 PMC5032908 27560171

[49] Patro R Duggal G Love MI Irizarry RA Kingsford C. Salmon provides fast and bias-aware quantification of transcript expression. Nat Methods (2017) 14(4):417–9. doi: 10.1038/nmeth.4197 PMC5600148 28263959

[50] Love MI Huber W Anders S. Moderated estimation of fold change and dispersion for RNA-seq data with DESeq2. Genome Biol (2014) 15(12):550. doi: 10.1186/s13059-014-0550-8 25516281 PMC4302049

[51] Wu T Hu E Xu S Chen M Guo P Dai Z. clusterProfiler 4.0: a universal enrichment tool for interpreting omics data. Innov Camb Mass (2021) 2(3):100141. doi: 10.1016/j.xinn.2021.100141 PMC8454663 34557778

[52] Gillespie M Jassal B Stephan R Milacic M Rothfels K Senff-Ribeiro A. The reactome pathway knowledgebase 2022. Nucleic Acids Res (2022) 50(D1):D687–D692. doi: 10.1093/nar/gkab1028 PMC8689983 34788843

[53] Kolde R. Pheatmap (2023). Available at: https://github.com/raivokolde/pheatmap.

[54] Waskom ML. Seaborn: statistical data visualization. J Open Source Software (2021) 6(60):3021. doi: 10.21105/joss.03021

[55] Guo Q Yang C Gao F. The state of CD44 activation in cancer progression and therapeutic targeting. FEBS J (2022) 289(24):7970–86. doi: 10.1111/febs.16179 34478583

[56] Mi Z Guo H Russell MB Liu Y Sullenger BA Kuo PC. RNA Aptamer blockade of osteopontin inhibits growth and metastasis of MDA-MB231 breast cancer cells. Mol Ther J Am Soc Gene Ther (2009) 17(1):153–61. doi: 10.1038/mt.2008.235 PMC2834992 18985031

[57] Talbot LJ Mi Z Bhattacharya SD Kim V Guo H Kuo PC. Pharmacokinetic characterization of an RNA aptamer against osteopontin and demonstration of *in vivo* efficacy in reversing growth of human breast cancer cells. Surgery (2011) 150(2):224–30. doi: 10.1016/j.surg.2011.05.015 PMC3148491 21801960

[58] Castello LM Raineri D Salmi L Clemente N Vaschetto R Quaglia M. Osteopontin at the crossroads of inflammation and tumor progression. Mediators Inflamm (2017) 2017:4049098. doi: 10.1155/2017/4049098 28769537 PMC5523273

[59] Hur EM Youssef S Haws ME Zhang SY Sobel RA Steinman L. Osteopontin-induced relapse and progression of autoimmune brain disease through enhanced survival of activated T cells. Nat Immunol (2007) 8(1):74–83. doi: 10.1038/ni1415 17143274

[60] Rangaswami H Bulbule A Kundu GC. Osteopontin: role in cell signaling and cancer progression. Trends Cell Biol (2006) 16(2):79–87. doi: 10.1016/j.tcb.2005.12.005 16406521

[61] Chambers AF Wilson SM Kerkvliet N O’Malley FP Harris JF Casson AG. Osteopontin expression in lung cancer. Lung Cancer Amst Neth (1996) 15(3):311–23. doi: 10.1016/0169-5002(95)00595-1 8959677

[62] Thalmann GN Sikes RA Devoll RE Kiefer JA Markwalder R Klima I. Osteopontin: possible role in prostate cancer progression. Clin Cancer Res (1999) 5(8):2271–7. 10473115

[63] Gotoh M Sakamoto M Kanetaka K Chuuma M Hirohashi S. Overexpression of osteopontin in hepatocellular carcinoma. Pathol Int (2002) 52(1):19–24. doi: 10.1046/j.1440-1827.2002.01316.x 11940202

[64] Irby RB McCarthy SM Yeatman TJ. Osteopontin regulates multiple functions contributing to human colon cancer development and progression. Clin Exp Metastasis (2004) 21(6):515–23. doi: 10.1007/s10585-004-2873-4 15679049

[65] Zhou Y Dai DL Martinka M Su M Zhang Y Campos EI. Osteopontin expression correlates with melanoma invasion. J Invest Dermatol (2005) 124(5):1044–52. doi: 10.1111/j.0022-202X.2005.23680.x 15854047

[66] Cook AC Tuck AB McCarthy S Turner JG Irby RB Bloom GC. Osteopontin induces multiple changes in gene expression that reflect the six “hallmarks of cancer” in a model of breast cancer progression. Mol Carcinog (2005) 43(4):225–36. doi: 10.1002/mc.20105 15864800

[67] Bandopadhyay M Bulbule A Butti R Chakraborty G Ghorpade P Ghosh P. Osteopontin as a therapeutic target for cancer. Expert Opin Ther Targets (2014) 18(8):883–95. doi: 10.1517/14728222.2014.925447 24899149

[68] Kariya Y Kariya Y. Osteopontin in cancer: mechanisms and therapeutic targets. Int J Transl Med (2022) 2(3):419–47. doi: 10.3390/ijtm2030033

[69] Pantazopoulos I Boura P Xanthos T Syrigos K. Effectiveness of mesothelin family proteins and osteopontin for malignant mesothelioma. Eur Respir J (2013) 41(3):706–15. doi: 10.1183/09031936.00226111 22835614

[70] Cristaudo A Foddis R Bonotti A Simonini S Vivaldi A Guglielmi G. Comparison between plasma and serum osteopontin levels: usefulness in diagnosis of epithelial malignant pleural mesothelioma. Int J Biol Markers (2010) 25(3):164–70. doi: 10.1177/172460081002500307 20878622

[71] Paleari L Rotolo N Imperatori A Puzone R Sessa F Franzi F. Osteopontin is not a specific marker in malignant pleural mesothelioma. Int J Biol Markers (2009) 24(2):112–7. doi: 10.1177/172460080902400208 19634115

[72] Lin H Shen YC Long HY Wang H Luo ZY Wei ZX. Performance of osteopontin in the diagnosis of malignant pleural mesothelioma: a meta-analysis. Int J Clin Exp Med (2014) 7(5):1289–96. PMC4073746 24995085

[73] Solinas G Schiarea S Liguori M Fabbri M Pesce S Zammataro L. Tumor-conditioned macrophages secrete migration-stimulating factor: a new marker for M2-polarization, influencing tumor cell motility. J Immunol Baltim Md 1950 (2010) 185(1):642–52. doi: 10.4049/jimmunol.1000413 20530259

[74] Rittling SR. Osteopontin in macrophage function. Expert Rev Mol Med (2011) 13:e15. doi: 10.1017/S1462399411001839 21545755

[75] Tan Y Zhao L Yang YG Liu W. The role of osteopontin in tumor progression through tumor-associated macrophages. Front Oncol (2022) 12:953283. doi: 10.3389/fonc.2022.953283 35898884 PMC9309262

[76] Zhu B Suzuki K Goldberg HA Rittling SR Denhardt DT McCulloch CAG. Osteopontin modulates CD44-dependent chemotaxis of peritoneal macrophages through G-protein-coupled receptors: evidence of a role for an intracellular form of osteopontin. J Cell Physiol (2004) 198(1):155–67. doi: 10.1002/jcp.10394 14584055

[77] Rao G Wang H Li B Huang L Xue D Wang X. Reciprocal interactions between tumor-associated macrophages and CD44-positive cancer cells via osteopontin/CD44 promote tumorigenicity in colorectal cancer. Clin Cancer Res Off J Am Assoc Cancer Res (2013) 19(4):785–97. doi: 10.1158/1078-0432.CCR-12-2788 23251004

[78] Chen C Zhao S Karnad A Freeman JW. The biology and role of CD44 in cancer progression: therapeutic implications. J Hematol OncolJ Hematol Oncol (2018) 11(1):64. doi: 10.1186/s13045-018-0605-5 29747682 PMC5946470

[79] Orian-Rousseau V Ponta H. Perspectives of CD44 targeting therapies. Arch Toxicol (2015) 89(1):3–14. doi: 10.1007/s00204-014-1424-2 25472903

[80] Cortes-Dericks L Schmid RA. CD44 and its ligand hyaluronan as potential biomarkers in malignant pleural mesothelioma: evidence and perspectives. Respir Res (2017) 18(1):58. doi: 10.1186/s12931-017-0546-5 28403901 PMC5389171

[81] Asplund T Heldin P. Hyaluronan receptors are expressed on human malignant mesothelioma cells but not on normal mesothelial cells. Cancer Res (1994) 54(16):4516–23. 7519123

[82] Penno MB Askin FB Ma H Carbone M Vargas MP Pass HI. High CD44 expression on human mesotheliomas mediates association with hyaluronan. Cancer J Sci Am (1995) 1(3):196–203. 9166476

[83] Sakurai Y Kato A Hida Y Hamada J Maishi N Hida K. Synergistic enhancement of cellular uptake with CD44-expressing malignant pleural mesothelioma by combining cationic liposome and hyaluronic acid-lipid conjugate. J Pharm Sci (2019) 108(10):3218–24. doi: 10.1016/j.xphs.2019.06.012 31229434

[84] Raineri D Dianzani C Cappellano G Maione F Baldanzi G Iacobucci I. Osteopontin binds ICOSL promoting tumor metastasis. Commun Biol (2020) 3(1):1–15. doi: 10.1038/s42003-020-01333-1 33106594 PMC7588454

[85] Raineri D Cappellano G Vilardo B Maione F Clemente N Canciani E. Inducible T-cell costimulator ligand plays a dual role in melanoma metastasis upon binding to osteopontin or inducible T-cell costimulator. Biomed (2022) 10(1):51. doi: 10.3390/biomedicines10010051 PMC8772802 35052731

[86] Hutloff A Dittrich AM Beier KC Eljaschewitsch B Kraft R Anagnostopoulos I. ICOS is an inducible T-cell co-stimulator structurally and functionally related to CD28. Nature (1999) 397(6716):263–6. doi: 10.1038/16717 9930702

[87] Fontecedro AC Cecconi V Bosco EF Schmitt-Opitz I Weder W Stahel RA. Chemotherapy of malignant pleural mesothelioma does not preclude use of check-point blockade. Ann Oncol (2015) 26:i48. doi: 10.1093/annonc/mdv052.01

