# Supplementary figures and images for: Important functional role of the protein osteopontin in the progression of malignant pleural mesothelioma
Source: Front Immunol. 2023 Jun 16;14:1116430. doi: 10.3389/fimmu.2023.1116430 (PMC10312076; doi:10.3389/fimmu.2023.1116430)

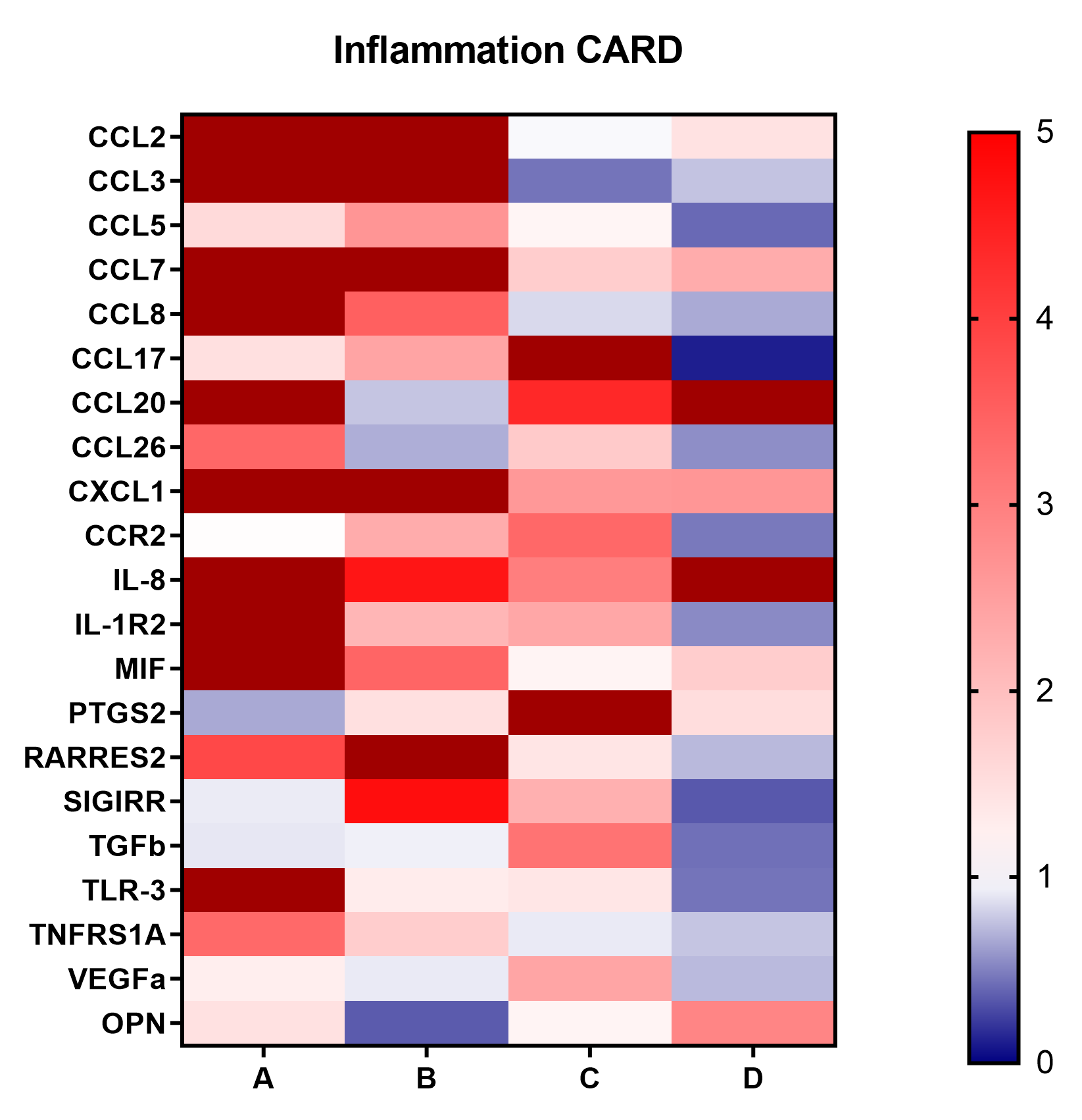

Supplement: Supplementary Figure 1 — Transcriptomic analysis of inflammatory genes in human malignant mesothelioma. Gene expression profiling of four surgical human MPMs samples using TaqMan Low Density Array containing inflammatory 91 genes. Data are shown as fold increase in tumor samples relative to the non-involved pleural tissue from each paired patient. Selected genes are shown for which at least 2 samples showed upregulation over normal tissues. [file Image_1.tif]

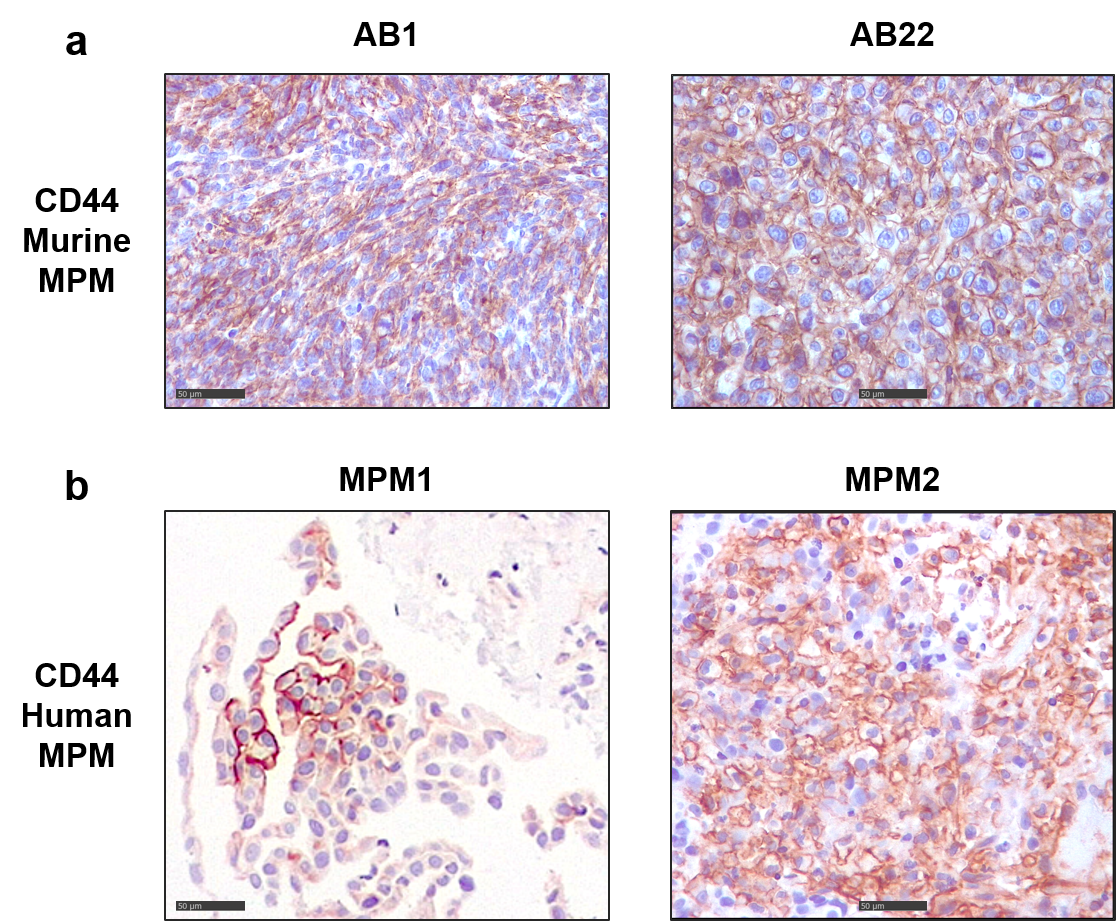

Supplement: Supplementary Figure 2 — Immunohistochemistry of CD44 expression by murine and human mesothelioma. a) murine mesothelioma tumors (AB1 and AB22) grown in vivo in mice. b) MPM1 and MPM2 are human mesothelioma surgical samples. [file Image_2.tif]

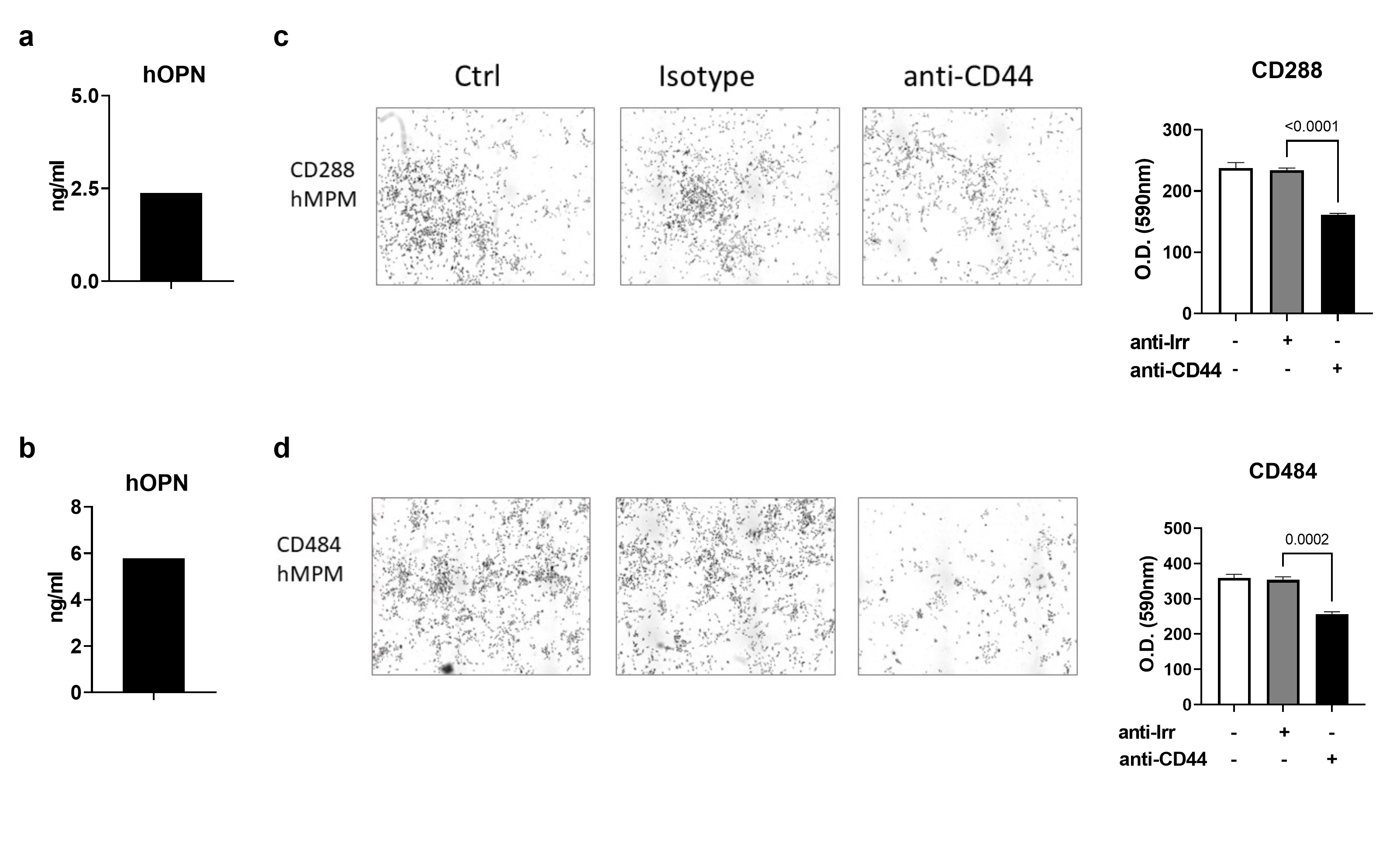

Supplement: Supplementary Figure 3 — In vitro characterization of hMPM cell lines. (A, B) ELISA for hOPN spontaneously produced by the cell lines CD288 and CD484 (epithelioid phenotype). (C, D) Representative images of colony assays and relative quantification: addition of a blocking anti-CD44 mAb (5 mg/ml) inhibits cell proliferation. Data are shown as mean +/- SD (One-way ANOVA). [file Image_3.tif]

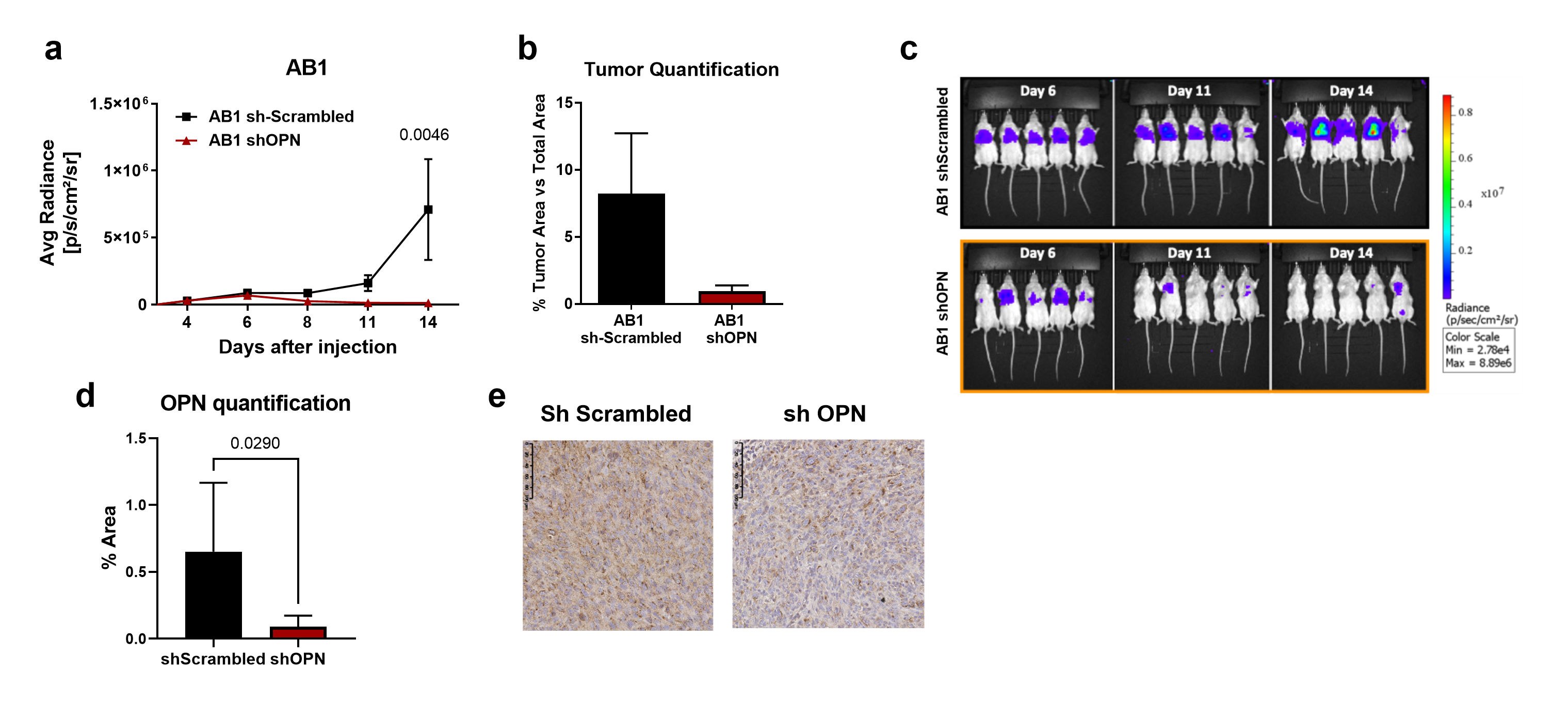

Supplement: Supplementary Figure 4 — Silencing of OPN impairs the growth of murine AB1 mesothelioma cells in vivo. a-b-c) In vivo growth of 5x10^4^ AB1 sh-scrambled or AB1 shOPN, injected intra-thoracically in BALB/c mice. (A) IVIS in vivo imaging luminescence signal, mean+/-SEM values of 5 mice; (B) Histological quantification of total tumor area. (C) Representative images of IVIS acquisition of LUC signal at different time points. (D, E) Immunohistochemistry of explanted tumors, relative quantification for the staining of OPN and representative pictures. Data are shown as mean +/- SEM (A Two-way ANOVA; B, D: Unpaired t-test with Welsh correction). [file Image_4.tif]

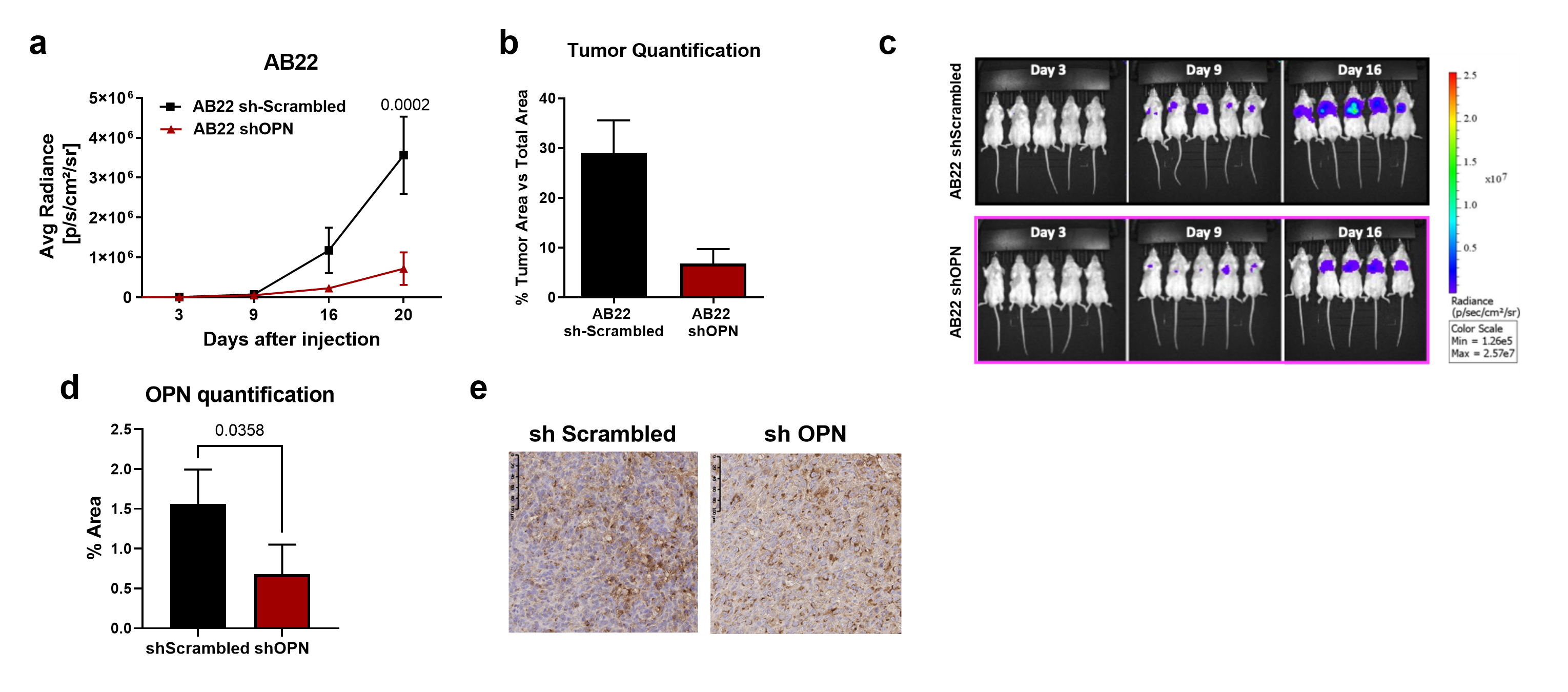

Supplement: Supplementary Figure 5 — Silencing of OPN impairs the growth of murine AB22 mesothelioma cells in vivo. (A–C) In vivo growth of 5x10^4^ AB22 sh-scrambled or AB22 shOPN, injected intra-thoracically in BALB/c mice. (A) IVIS in vivo imaging luminescence signal, mean+/-SEM values of 5 mice; (B) Histological quantification of total tumor area. (C) Representative images of IVIS acquisition of LUC signal at different time points. (D, E) Immunohistochemistry of explanted tumors, relative quantification for the staining of OPN and representative pictures. Data are shown as mean +/- SEM (A Two-way ANOVA; B, D: Unpaired t-test with Welsh correction). [file Image_5.tif]

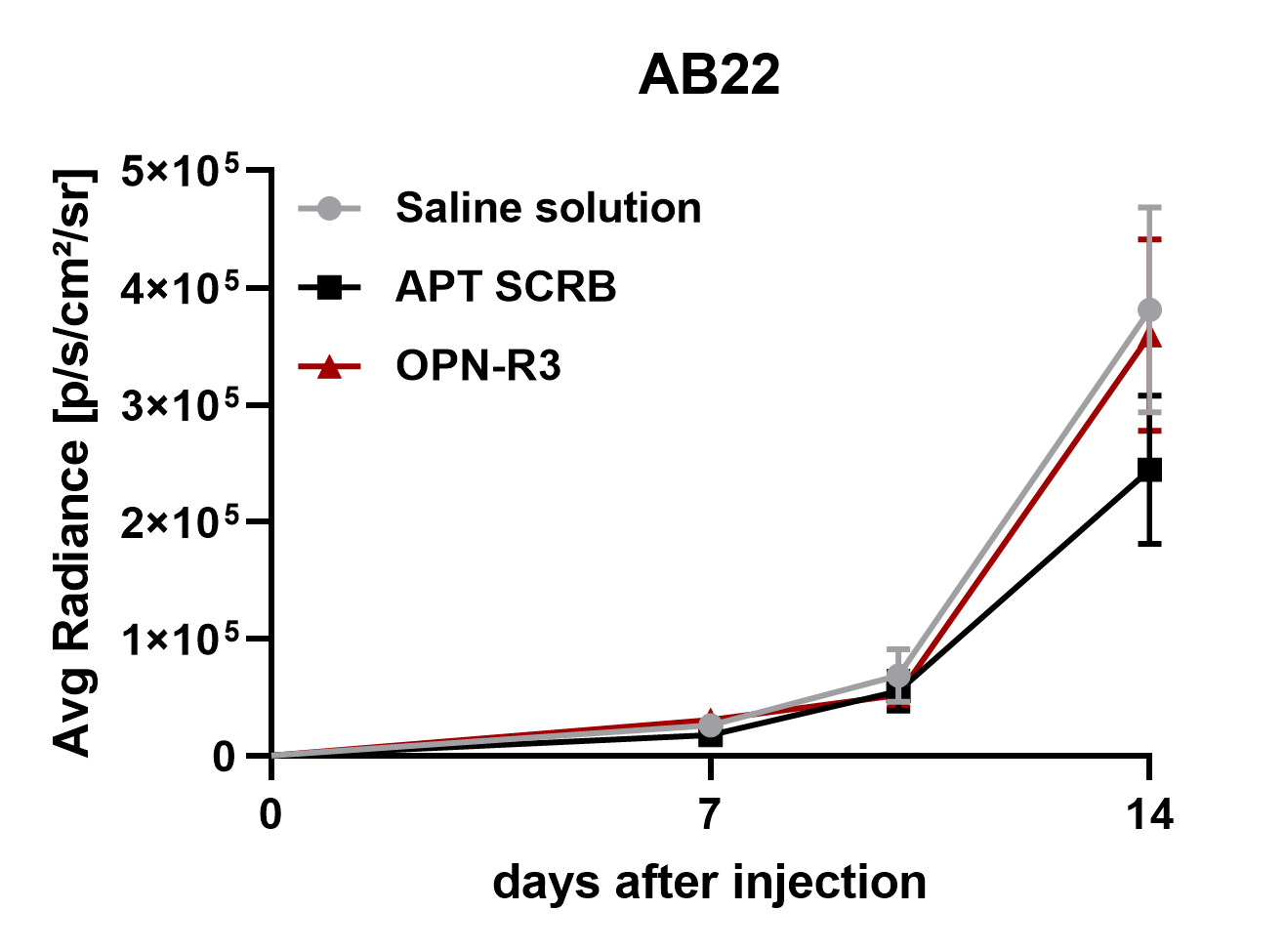

Supplement: Supplementary Figure 6 — Inhibition of OPN with the aptamer OPN-R3 does not affect the in vivo growth of murine AB22 mesothelioma cells. Results of IVIS in vivo imaging luminescence signal, mean+/-SEM values of 5 mice per group. [file Image_6.tif]

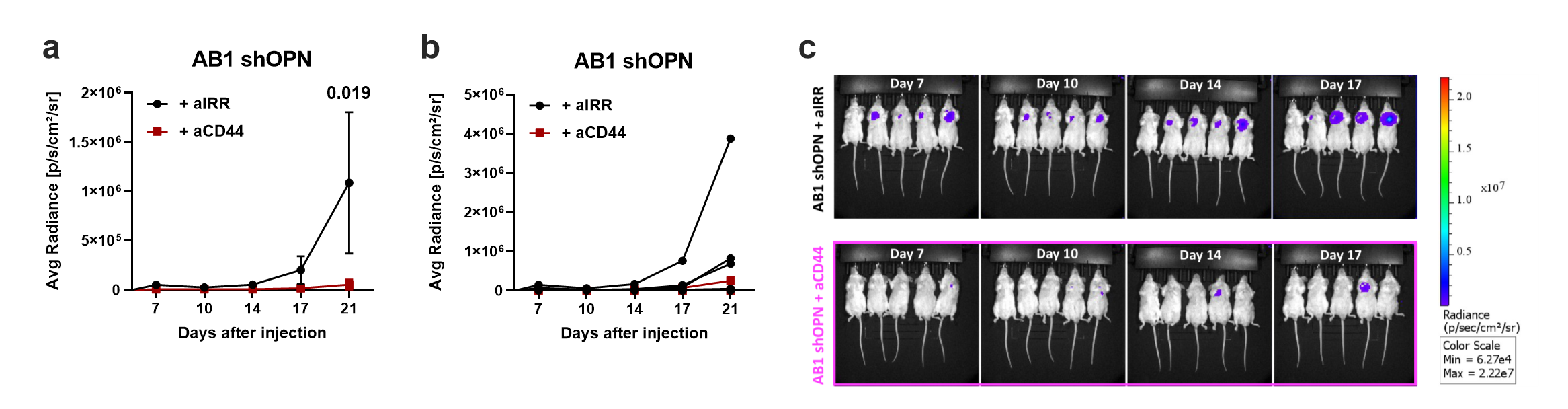

Supplement: Supplementary Figure 7 — Treatment with blocking anti-CD44 mAbs impairs in vivo growth of murine mesothelioma cells. (A, C) Effect of anti-CD44 mAbs on AB1 shOPN tumor growth. Mice were treated intra-peritoneally with anti-CD44 (10 mg/kg) or with irrelevant mAbs at day (4, 7, 12, 16, 19) post tumor injection. Data are expressed as average radiance, (A) mean+/-SEM values of 5 mice; (B) values of single mice; (C) Representative images of IVIS acquisition of LUC signal at different time points. [file Image_7.tif]
